# Associations between neutrophil-lymphocyte ratio and monocyte to high-density lipoprotein ratio with left atrial spontaneous echo contrast or thrombus in patients with non-valvular atrial fibrillation

**DOI:** 10.1186/s12872-023-03270-3

**Published:** 2023-05-04

**Authors:** Yingjian Deng, Faguang Zhou, Qiang Li, Jincun Guo, Binni Cai, Guiyang Li, Jianghai Liu, Linlin Li, Qi Zheng, Dong Chang

**Affiliations:** 1 Department of Cardiology, School of Medicine, Xiamen Cardiovascular Hospital of Xiamen University, Xiamen University, Xiamen, China; 2 Department of Psychology, Xiamen Xianyue Hospital, Xiamen, China

**Keywords:** Atrial fibrillation, Stroke, Left atrial appendage thrombus, Spontaneous echo contrast, Neutrophil-lymphocyte ratio, Monocyte to high-density lipoprotein ratio

## Abstract

**Background:**

The importance of inflammation in thrombosis is increasingly appreciated. Neutrophil-lymphocyte ratio (NLR) and monocyte to high-density lipoprotein ratio (MHR) are important indicators of systemic inflammation. This study aimed to investigate the associations between NLR and MHR with left atrial appendage thrombus (LAAT) and spontaneous echo contrast (SEC) in patients with non-valvular atrial fibrillation.

**Methods:**

This retrospective, cross-sectional study enrolled 569 consecutive patients with non-valvular atrial fibrillation. Multivariable logistic regression analysis was used to investigate independent risk factors of LAAT/SEC. Receiver operating characteristic (ROC) curves were used to evaluate the specificity and sensitivity of NLR and MHR in predicting LAAT/SEC. Subgroup and Pearson correlation analyses were used to assess the correlations between NLR and MHR with the CHA_2_DS_2_-VASc score.

**Results:**

Multivariate logistic regression analysis showed that NLR (OR: 1.49; 95%CI: 1.173–1.892) and MHR (OR: 2.951; 95%CI: 1.045–8.336) were independent risk factors for LAAT/SEC. The area under the ROC curve of NLR (0.639) and MHR (0.626) was similar to that of the CHADS_2_ score (0.660) and CHA_2_DS_2_-VASc score (0.637). Subgroup and Pearson correlation analyses showed significant but very weak associations between NLR (r = 0.139, *P* < 0.05) and MHR (r = 0.095, *P* < 0.05) with the CHA_2_DS_2_-VASc score.

**Conclusion:**

Generally, NLR and MHR are independent risk factors for predicting LAAT/SEC in patients with non-valvular atrial fibrillation.

## Introduction

Atrial fibrillation (AF) is the most common and clinically significant persistent cardiac arrhythmia, with a prevalence of 1–3% in the general population that increased with age [1, 2]. AF is associated with a fivefold increased risk for stroke, leading to high morbidity and mortality [3, 4]. Anticoagulant therapy has been recommended as the most efficacious way to reduce the risk of stroke among patients with AF by nearly 60%, according to a meta-analysis [5]. Thus, early identification of thrombosis and signs of risk is of paramount importance to initiate timely anticoagulant therapy for stroke prevention. The left atrium (LA) and left atrial appendage (LAA) are the primary sites of thrombosis, with over 90% of embolic strokes reported to be caused by left atrial appendage thrombosis (LAAT) [6]. Spontaneous echo contrast (SEC), defined as the echogenicity of blood in the absence of contrast agents, is another well-recognized precursor for thrombosis that indicates blood stasis [7]. The most sensitive method for the detection of LAAT and SEC is transesophageal echocardiography (TEE) [8, 9]. However, this invasive examination may increase the risk of complications such as esophageal trauma, causing an additional burden on the patients. Since LAAT and SEC are both preventable and treatable, a safer and non-invasive assessment method is needed to detect LAAT/SEC to guide early anticoagulant treatment and stroke prevention.

In previous studies, the CHADS_2_ and CHA_2_DS_2_-VASc scores are mostly used to assess the risk of stroke; however, recent studies also investigated their value in predicting LAAT/SEC [10]. Although widely recommended for the most of risks of stroke in AF patients, the above prediction models showed inconsistent performances in subsequent validation studies. For instance, A recent systematic review and meta-analysis suggested that the power of the CHA_2_DS_2_-VASc score in the prediction of stroke is modest, highlighting the need for models with higher accuracy [11]. Previous studies also showed only modest predictive performances of these conventional scoring systems for LAAT/SEC prediction [12, 13]. Another major limitation of the CHADS_2_ and CHA_2_DS_2_-VASc score systems is the lack of inclusion of other potential risk factors for stroke, such as cancer [14], arthritis [15], rheumatic disease [16], and chronic kidney disease [17], all of which indicate the presence of inflammation. Inflammation and thrombosis are closely connected processes, and growing evidence has shown that inflammation might play a critical role in the development of LAAT/SEC [18–20]. It is thus expected that some novel predictive biomarkers for inflammation may help refine the current stroke risk assessment system by providing a more accurate prediction of LAAT/SEC in patients with AF.

Neutrophil-lymphocyte ratio (NLR) and monocyte to high-density lipoprotein ratio (MHR) are inexpensive and easily obtained biomarkers for systemic inflammation, which leads to an increased risk of stroke and mortality [21, 22]. Beomseok et al. found that a high level of NLR is an independent risk factor for ischemic stroke in healthy individuals, indicating the possibility of reclassification for stroke incidence in patients with AF [23]. A recent study also suggested that NLR might be a beneficial predictor for the potential acute venous thromboembolism (VTE) [24]. It has been found that high-density lipoprotein cholesterol (HDL-C) could suppress the pro-inflammatory and pro-oxidant effects of monocytes [25] and that it also has anti-inflammatory, antioxidant, and anti-thrombotic effects [26]. Therefore, decreased HDL-C and increased monocytes, reflected as increased MHR, may be an indicator of inflammation. In recent years, NLR and MHR have been widely reported to be risk factors for cardiovascular diseases and to be associated with increased all-cause mortality [27–29]. Some studies also indicated that NLR and MHR might be associated with thromboembolic stroke in patients with non-valvular AF (NVAF) [30–32]. Although both NLR [33, 34] and MHR [35] have been well established to be predictive of new-onset AF and the risk of stroke, previous studies on inflammation biomarkers mostly focused on their associations with ischemic stroke in patients with AF rather than those at high risk but with no stroke. In addition, stroke risk stratification could be confounded by non-cardioembolic stroke, misleading the anticoagulant treatment for patients with AF. As a result, it might be more informative to establish the association between NLR/MHR and the more specific LAAT/SEC, instead of the general risk of stroke, to avoid confounding. To our knowledge, there has been no study exploring the value of NLR and MHR in the prediction of LAAT/SEC in patients with NVAF. Therefore, we conducted the current study to evaluate the associations between NLR and MHR with LAAT/SEC and their correlations with the CHA_2_DS_2_-VASc score.

## Materials and methods

In this retrospective cross-sectional study, we aimed to investigate the associations between NLR and MHR with LAAT/SEC based on data available in the medical records of consecutive patients with NVAF who were admitted to the Xiamen Cardiovascular Hospital for radiofrequency catheter ablation from June 2019 to May 2021.

A total of 569 eligible patients diagnosed with NVAF who underwent TEE were enrolled in this study and assigned into two groups: the LAAT/SEC group with a diagnosis of LAAT or SEC as confirmed by TEE (n = 98), and a negative control group (n = 471). Patients with leukemia, recent allergy or infection, and liver dysfunction were excluded. Patients with heart diseases including valvular heart disease, acute myocardial infarction, rheumatic heart disease, and those on medications that might affect the complete blood count were also excluded. The study complied with the principles of the Declaration of Helsinki and was approved by the Ethics Committee of Xiamen Cardiovascular Hospital. All procedures in this study were performed in accordance with the institutional guidelines.

Demographic and clinical data of the patients were collected after admission. All the subjects were tested for complete blood count and lipid profile. NLR was calculated as the absolute neutrophil count divided by the absolute lymphocyte count, and MHR was calculated as the monocyte count divided by the level of HDL. LAAT/SEC was diagnosed through TEE performed by experienced sonographers. LAAT was defined as well-circumscribed, echogenic masses with a different texture but uniform consistency, as compared with the LA wall. SEC was defined as a dynamic smoke-like signal with a swirling pattern in the LA and LAA, which could be detected by excessive gain under appropriate gain settings [36]. The CHADS_2_ and CHA_2_DS_2_-VASc scores were calculated based on their respective indicator scores, and the patients were further divided into the following four subgroups based on their CHA_2_DS_2_-VASc scores: 0 (Group 1), 1 (Group 2), 2–4 (Group 3), and ≥ 5 (Group 4).

### Statistical analysis

Statistical analyses were performed using the SPSS software (version 22.0). Normally distributed continuous data were presented as mean ± standard deviation (SD), and inter-group comparisons were performed using independent samples t-test or one-way ANOVA as appropriate. Possible risk factors of LAAT/SEC were analyzed using multivariate logistic regression analysis. Model 1 included all risk factors with *P* < 0.05 in univariate analysis, while Model 2 and Model 3 only included NLR, MHR, and the CHADS_2_ score or the CHA_2_DS_2_-VASc score. Receiver operating characteristic (ROC) curve analysis was performed to evaluate the specificity and sensitivity of NLR and MHR for the prediction of LAAT/SEC. The cut-off score was selected as the point that maximized both sensitivity and specificity. Correlations of NLR and MHR with CHA_2_DS_2_-VASc scores were evaluated using the Pearson correlation coefficient (r) and visualized using a scatter plot. *P* < 0.05 indicated statistically significant differences.

## Results

A total of 569 patients were included in this study, with 98 (17.2%) patients in the LAAT/SEC group and 471 (82.8%) in the control group. The clinical characteristics of the patients are summarized in Table 1. Significant differences between the LAAT/SEC group and the control group were found in NLR, MHR, CHADS_2_ score, and CHA_2_DS_2_-VASc score, as well as other clinical indicators such as white blood cell (WBC) count, neutrophil count, monocyte count, HDL, estimated glomerular filtration (eGFR), left atrial diameter (LAD), ejection fraction (EF), heart failure, previous stroke/transient ischemic attack (TIA), peripheral arterial disease, and chronic kidney disease (*P* < 0.05) (Table 1). As many of the patients were not on any anticoagulant drug before admission, the impact of anticoagulants could not be determined.

**Table 1:** Baseline characteristics of the two groups

Variables	The LAAT/SEC group	The control group	*P* value
	**N = 98**	** N = 471**	
Age, years	64.08 ± 10.65	61.68 ± 11.67	0.060
Female, n (%)	33 (33.67)	166 (35.24)	0.767
Laboratory data			
WBC count, 10^9^/L	7.00 ± 1.93	6.47 ± 1.35	0.001*
HB, g/L	140.25 ± 17.60	139.47 ± 14.89	0.652
Platelet count, 10^9^/L	208.19 ± 47.13	209.35 ± 51.11	0.837
MPV, fL	9.21 ± 1.06	9.04 ± 0.94	0.121
Neutrophil count, 10^9^/L	4.68 ± 1.66	3.98 ± 0.99	< 0.001*
Lymphocyte count, 10^9^/L	1.74 ± 0.63	1.91 ± 0.88	0.066
Monocyte count, 10^9^/L	0.56 ± 0.72	0.43 ± 0.25	0.002*
HDL, mmol/L	1.05 ± 0.27	1.12 ± 0.27	0.018*
Creatinine, µmol/L	84.18 ± 27.25	80.47 ± 46.45	0.446
Uric acid, µmol/L	409.09 ± 123.39	393.87 ± 288.69	0.609
eGFR, ml/ (min 1.73 m^2^)	79.57 ± 22.72	87.26 ± 29.51	0.015*
Echocardiographic parameters			
LAD, mm	44.47 ± 5.86	39.30 ± 5.39	< 0.001*
LVEDD, mm	48.46 ± 6.22	47.57 ± 5.36	0.149
EF, %	59.45 ± 9.93	63.63 ± 8.37	< 0.001*
Medical history			
Hypertension, n (%)	54 (55.10)	233 (49.47)	0.310
Diabetes mellitus, n (%)	25 (25.51)	92 (19.53)	0.183
Coronary heart disease, n (%)	23 (23.47)	82 (17.41)	0.159
Heart failure, n (%)	45 (45.92)	68 (14.44)	< 0.001*
Previous stroke/TIA, n (%)	16 (16.33)	24 (5.10)	< 0.001*
Peripheral arterial disease, n (%)	10 (10.20)	12 (2.55)	0.001*
Chronic kidney disease, n (%)	10 (10.20)	22 (4.67)	0.031*
Hyperthyroidism, n (%)	8 (8.16)	31 (6.58)	0.573
Medication			
Dabigatran, n (%)	27 (27.55)	183 (38.85)	0.035*
Rivaroxaban, n (%)	60 (61.22)	242 (51.38)	0.076
Warfarin, n (%)	12 (12.24)	36 (7.64)	0.136
Antiplatelet, n (%)	8 (8.16)	11 (2.34)	0.009*
NLR	3.05 ± 1.67	2.31 ± 0.88	< 0.001*
MHR	0.54 ± 0.53	0.40 ± 0.21	< 0.001*
CHADS_2_ Score	1.96 ± 1.47	1.20 ± 1.33	< 0.001*
CHA_2_DS_2_-VASc Score	2.88 ± 1.74	2.06 ± 1.63	< 0.001*

Abbreviations: WBC: white blood cell; HB: hemoglobin; MPV: mean platelet volume; HDL: high-density lipoprotein; eGFR: estimated glomerular filtration rate; LAD: left atrial diameter; LVEDD: left ventricular end-diastolic diameter; EF: ejection fraction; TIA: transient ischemic attack; NLR: neutrophil-lymphocyte ratio; MHR: monocyte to high-density lipoprotein ratio; *: with statistical significance

In multivariable logistic regression analysis (Model 1), all risk factors with *P* < 0.05 in univariate analysis were included, which showed that heart failure (OR: 3.876; 95%CI: 1.876–8.007), previous stroke/TIA (OR: 4.079; 95%CI: 1.417–11.743), LAD (OR: 1.148; 95%CI: 1.09–1.209), NLR (OR: 1.49; 95%CI: 1.173–1.892) and MHR (OR: 2.951; 95%CI: 1.045–8.336) were independent risk factors for LAAT/SEC (Table 2). However, neither the CHADS_2_ score nor the CHA_2_DS_2_-VASc score was statistically significantly associated with LAAT/SEC after controlling for other variables. Model 2 and Model 3 only included NLR, MHR, and the CHADS_2_ score or the CHA_2_DS_2_-VASc score, which showed that the CHADS_2_ score and CHA_2_DS_2_-VASc score were independent risk factors for LAAT/SEC after adjusting for NLR and MHR (Table 2).

**Table 2:** Logistic regression analysis of the risk factors for LAAT/SEC

Variables	β	SE	Wald	OR (95%CI)	*p* value
**Model 1**					
WBC count	0.092	0.094	0.968	1.096 (0.913, 1.317)	0.325
eGFR	-0.005	0.006	0.789	0.995 (0.984, 1.006)	0.374
Heart failure	1.355	0.370	13.390	3.876 (1.876, 8.007)	< 0.001*
Previous stroke/TIA	1.406	0.540	6.790	4.079 (1.417, 11.743)	0.009*
Peripheral arterial disease	0.839	0.590	2.022	2.315 (0.728, 7.36)	0.155
Chronic kidney disease	0.041	0.525	0.006	1.041 (0.372, 2.917)	0.938
LAD	0.138	0.026	27.600	1.148 (1.09, 1.209)	< 0.001*
EF	0.011	0.016	0.499	1.011 (0.98, 1.043)	0.480
NLR	0.399	0.122	10.700	1.49 (1.173, 1.892)	0.001*
MHR	1.082	0.53	4.173	2.951 (1.045, 8.336)	0.041*
CHADS_2_ Score	-0.034	0.211	0.026	0.967 (0.639, 1.461)	0.872
CHA_2_DS_2_-VASc Score	-0.116	0.171	0.459	0.89 (0.636, 1.246)	0.498
**Model 2**					
NLR	0.520	0.107	23.664	1.682(1.364, 2.074)	< 0.001*
MHR	1.375	0.476	8.356	3.955(1.557, 10.046)	0.004*
CHADS_2_ Score	0.284	0.078	13.195	1.328(1.139, 1.547)	< 0.001*
**Model 3**					
NLR	0.523	0.107	23.829	1.688(1.368, 2.083)	< 0.001*
MHR	1.495	0.486	9.478	4.46(1.722, 11.553)	0.002*
CHA_2_DS_2_-VASc Score	0.224	0.068	10.896	1.251(1.095, 1.429)	0.001*

Abbreviations: WBC: white blood cell; eGFR: estimated glomerular filtration rate; TIA: transient ischemic attack; LAD: left atrial diameter; EF: ejection fraction; NLR: neutrophil-lymphocyte ratio; MHR: monocyte to high-density lipoprotein ratio; SE: standard error; *: with statistical significance

**Fig. 1:**
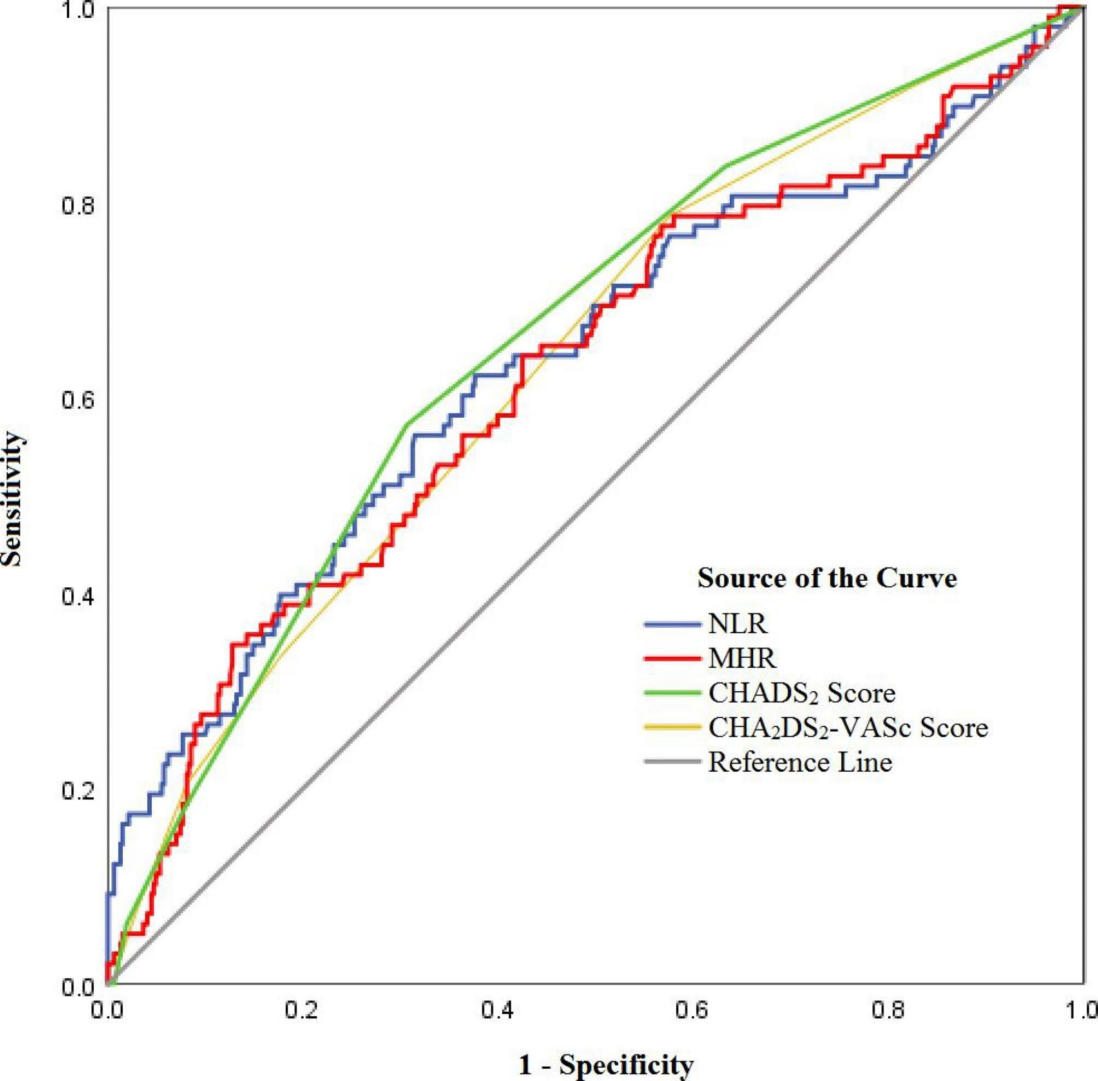
ROC curve analyses of NLR, MHR, CHADS_2_, and CHA_2_DS_2_-VASc in predicting LAAT/SEC

The ROC curve analyses of NLR, MHR, CHADS_2_ score and CHA_2_DS_2_-VASc score in the prediction of LAAT/SEC are presented in Fig. 1; Table 3. All four indicators demonstrated comparable discrimination ability in distinguishing the LAAT/SEC group from the control group. The area under the ROC curve (AUC) of NLR (AUC = 0.639) and MHR (AUC = 0.626) were similar to that of CHADS_2_ score (AUC = 0.660) and CHA_2_DS_2_-VASc score (AUC = 0.637). As for sensitivity and specificity in predicting LAAT/SEC, an NLR cut-off score of 2.57 showed a sensitivity of 0.561 and specificity of 0.686, while an MHR cut-off score of 0.566 showed a sensitivity of 0.347 and specificity of 0.873 (Table 3). Across the four indicators, the CHA_2_DS_2_-VASc score showed the highest sensitivity (0.786), and MHR showed the highest specificity (0.873) in the ROC curve analysis (Table 3).

**Table 3:** Prediction ability of NLR, MHR, CHADS_2_, and CHA_2_DS_2_-VASc for LAAT/SEC

Variables	AUC	SE	*P* value	95% CI	Cut-off	Sensitivity	Specificity
NLR	0.639	0.033	< 0.001	0.573 to 0.704	2.570	0.561	0.686
MHR	0.626	0.033	< 0.001	0.562 to 0.690	0.566	0.347	0.873
CHADS_2_ score	0.660	0.030	< 0.001	0.602 to 0.719	1.500	0.571	0.694
CHA_2_DS_2_-VASc score	0.637	0.030	< 0.001	0.577 to 0.696	1.500	0.786	0.425

Abbreviations: NLR: neutrophil-lymphocyte ratio; MHR: monocyte to high-density lipoprotein ratio; AUC: area under ROC curve; SE: standard error; ROC: receiver operating characteristic

The results of subgroup analyses on the prevalence of LAAT/SEC and levels of NLR and MHR according to the CHA_2_DS_2_-VASc score classification are presented in Table 4. Generally, there was an increasing trend of LAAT/SEC, NLR, and MHR with the CHA_2_DS_2_-VASc score. In the comparison between subgroups, the incidence of LAAT/SEC was significantly higher in group 3 (19.66%) and group 4 (34.48%), as compared with group 1 (8.79%) and group 2 (10.00%). The NLR was also significantly higher in group 3 (2.55 ± 1.15) and group 4 (2.69 ± 1.41), as compared with group 1 (2.27 ± 0.91) and group 2 (2.20 ± 0.84). The MHR was significantly higher in group 4 (0.55 ± 0.67) only, as compared with group 1 (0.42 ± 0.19), group 2 (0.41 ± 0.26), and group 3 (0.42 ± 0.19) (Table 4).

**Table 4:** Prevalence of LAAT/SEC and levels of NLR and MHR in different CHA_2_DS_2_-VASc groups

Variables	Group1	Group2	Group 3	Group 4	F/χ2	*P* value
	(n = 91)	(n = 130)	(n = 290)	(n = 58)		
LAAT/SEC	8 (8.79)	13 (10.00)	57 (19.66) *†	20 (34.48) *†£	22.618	< 0.001
NLR	2.27 ± 0.91	2.20 ± 0.84	2.55 ± 1.15*†	2.69 ± 1.41*†	4.844	0.002
MHR	0.42 ± 0.19	0.41 ± 0.26	0.42 ± 0.19	0.55 ± 0.67*†£	3.763	0.011

Group 1: CHA_2_DS_2_-VASc = 0; Group 2: CHA_2_DS_2_-VASc = 1; Group 3: CHA_2_DS_2_-VASc = 2–4; Group 4: CHA_2_DS_2_-VASc ≥ 5. *, †, £ each indicates significant difference compared with group 1, 2 and 3, respectively

**Fig. 2:**
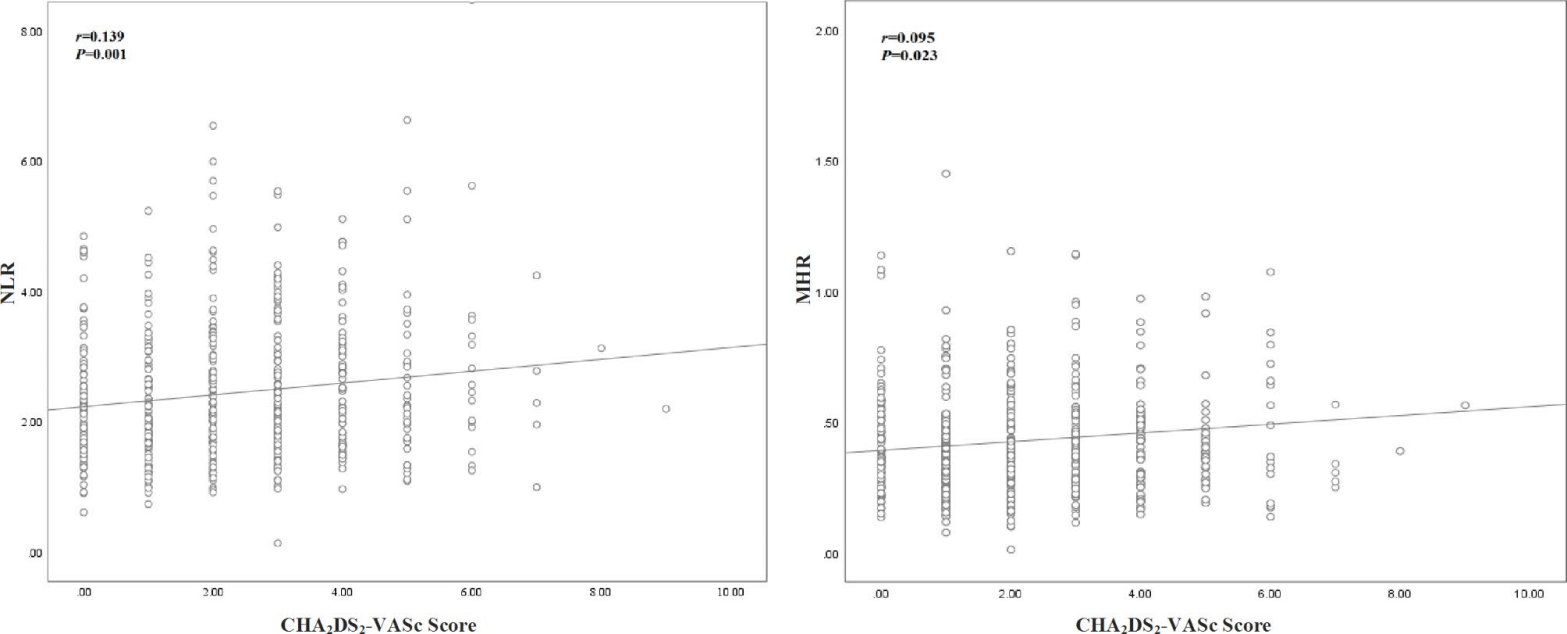
Correlation plots between NLR/MHR and CHA_2_DS_2_-VASc score

The correlations of NLR and MHR with the CHA_2_DS_2_-VASc score in patients with NVAF are presented in Fig. 2. Both NLR and MHR showed significant correlations with the CHA_2_DS_2_-VASc score, but the correlation coefficients were small (r = 0.139 and *P* < 0.05 for NLR; r = 0.095 and *P* < 0.05 for MHR).

## Discussion

In this cross-sectional study, we innovatively used two inflammation indicators, i.e., NLR and MHR, to investigate their associations with LAAT/SEC in patients with NVAF and evaluated their predictive performances as compared to the conventional CHADS_2_ and CHA_2_DS_2_-VASc score systems. Our major findings showed that both NLR and MHR are independent risk factors for predicting LAAT/SEC in patients with NVAF. Further subgroup and Pearson correlation analyses showed significant but very weak associations between NLR and MHR with the CHA_2_DS_2_-VASc score, suggesting their implication for the reclassification improvement of ischemic stroke in patients with NVAF.

Our major finding was that NLR and MHR were comparable and relatively independent from the conventional CHADS_2_ and CHA_2_DS_2_-VASc score systems in predicting LAAT/SEC among NVAF patients. NLR and MHR reflect the balance of neutrophils, lymphocytes, monocytes, and high-density lipoprotein in inflammatory and immune responses. The possible mechanism of NLR and MHR affecting thrombosis may include the following: (1) neutrophil extracellular traps (NETs) released by neutrophils play a critical role in the mechanisms underlying thrombosis, as demonstrated by recent studies [37–39]; (2) the majority of tissue factors associated with thrombosis are derived from monocytes [40], which also regulate the resolution of thrombus, with different monocyte subtypes playing different roles [41]; (3) lymphocytes have also been demonstrated to regulate the composition of thrombosis [42, 43]; and (4) HDL-C has anti-inflammatory, antioxidant, and anti-thrombotic effects, which may explain the underlying mechanisms on thrombosis, while low-density lipoprotein has been shown to promote thrombosis [44].

The associations between NLR and MHR with LAAT/SEC were consistent with previous evidence showing that inflammation was a contributing factor to AF and stroke [45–48]. Although it has been recognized that an inflammatory state can lead to coagulation, the underlying mechanism remains unclear. One possible explanation is that inflammation leads to a higher risk of thrombosis through upregulating procoagulant factors and downregulating anticoagulant factors and fibrinolytic activities [49]. In addition, inflammatory mediators may also increase platelet reactivities, leading to a prothrombotic state and thereby promoting thrombosis [50, 51]. In 2013, Engelmann et al. first used the term “immunothrombosis” to describe a physiological type of thrombosis in microvessels induced by immune cells and thrombosis-specific molecular mediators [52]. According to Engelmann et al., immunothrombosis involves a platform consisting of fibrin, neutrophils, monocytes, and platelets, which is also similar to large venous thrombosis [52]. On this basis, inflammation seems to be an important participant, rather than a bystander, in the process of thrombosis that leads to stroke. Although the CHADS_2_ or CHA_2_DS_2_-VASc score showed a significant association with LAAT/SEC in the univariate analysis, this association was insignificant in the subsequent multivariate regression when controlling for NLR, MHR, and other clinical factors. Further subgroup and Pearson correlation analyses showed very weak associations between NLR and MHR with CHA_2_DS_2_-VASc. These findings have further indicated the comparable and supplementary utility of NLR and MHR in stroke risk assessment above the conventional CHADS_2_ and CHA_2_DS_2_-VASc score systems.

Subgroup analyses based on the CHA_2_DS_2_-VASc score were performed to investigate the correlations between NLR and MHR and this conventional score model. The results showed that the incidence of LAAT/SEC and the levels of NLR and MHR were significantly higher in patients with higher CHA_2_DS_2_-VASc scores (≥ 2), which was consistent with findings in previous studies. Similarly, Gokhan et al. found that the CHADS_2_ score was significantly higher in the group with high NLR levels, suggesting that NLR might be a predictor of thromboembolic stroke in patients with NVAF [32]. Kahraman et al. suggested that NLR was related to the CHA_2_DS_2_-VASc score and was predictive of the risk of thromboembolism and hemorrhage [53]. However, to what extent NLR and MHR are related to the CHA_2_DS_2_-VASc score still remains unknown. Although both NLR and MHR showed significant correlations with the CHA_2_DS_2_-VASc score, the correlations were very weak, suggesting the possibility of relative independence of NLR and MHR from the CHA_2_DS_2_-VASc score. This result suggested that NLR and MHR, easily accessible clinical parameters, might further assist physicians to identify patients at high risk for stroke.

## Conclusion

In this study, we found that NLR and MHR were independent risk factors for predicting LAAT/SEC in patients with NVAF. NLR and MHR had comparable performances with the conventional CHADS_2_ score and CHA_2_DS_2_-VASc score in predicting LAAT/SEC while making up for the limitations of these conventional score systems. NLR and MHR may be used as alternative stroke risk stratification schemes in clinical practice.

## Data Availability

The datasets used during the current study are available from the corresponding author on reasonable request.
